# Who determines nasopharyngeal carcinoma inpatients’ medical expenditure? Importance factor ranking using the random forest model

**DOI:** 10.3389/fpubh.2025.1639687

**Published:** 2025-08-20

**Authors:** Pinghua Zhu, Yu Zhang, Lilian Huang, Bo Wei, Xiaoqiang Zhu, Pingping Zeng, Jingya Nong

**Affiliations:** ^1^School of Humanities and Social Science, Guangxi Medical University, Nanning, Guangxi, China; ^2^Affiliated Cancer Hospital of Guangxi Medical University, Nanning, Guangxi, China; ^3^Guangxi Maternal and Child Health Hospital, Nanning, Guangxi, China; ^4^Guangxi Hospital Association, Nanning, Guangxi, China; ^5^Nursing College, Guangxi Medical University, Nanning, Guangxi, China; ^6^School of Humanities and Social Science (School of Public Administration), Beihang University, Beijing, China

**Keywords:** nasopharyngeal carcinoma, total medical expenditure, out-of-pocket costs, the rates of out-of-pocket costs, random forest model

## Abstract

**Background:**

Nasopharyngeal carcinoma (NPC) is a malignant epithelial tumor most commonly in China. In 2013, NPC incidence and mortality in China were also at high levels worldwide, which poses a great health burden in China. This study analyzes the medical expenditure and influencing factors of inpatients with NPC and aims to provide reference suggestions for reducing medical expenditure for NPC.

**Methods:**

Based on the data from one of the western China hospitals, we use the random forest model to identify the important factors in total medical expenditure, out-of-pocket costs, and the rates of out-of-pocket costs.

**Results:**

Total medical expenditure, out-of-pocket costs, and the rates of out-of-pocket costs were decreased. According to the indicators of InNodePurti and %IncMSE, the top three influencing factors of total medical expenditure and out-of-pocket costs are Length of stay, Medical payment method, and Readmission status; the top three influencing factors of the rates of out-of-pocket costs are Medical payment method, Occupation, and Age.

**Conclusion:**

Length of stay was an important factor in total medical expenditure and out-of-pocket costs, and the medical payment method was an important factor in the rates of out-of-pocket costs. To reduce the burden of NPC patients’ medical expenditure, (1) we can reduce the length of stay by improving the level of medical technology; (2) increasing government medical expenditures and improving the level of medical security; (3)promoting smoking bans, and strengthening screening in high-risk areas.

## 1 Introduction

Nasopharyngeal carcinoma (NPC) is a malignant epithelial tumor most commonly arising from the Fossa of Rossenmueller (1). NPC has very distinct geographic areas of high risk, the highest rates are in populations in South-Eastern Asia (2), especially in China’s southern and eastern regions: Guangdong, Hainan, Guangxi, Hunan, and Fujian (3, 4). NPC is endemic to southern China, Southeast Asia, and Africa, with age-standardized incidence rates of 4–25 per 100,000 population in these regions, which are approximately 50–100 times higher than the incidence rates in the rest of the world (5). And an age-standardized rate (world) of 3.0 per 100,000 in China, which is about 7 times that the population mainly white (6). Over the last three decades, the age-standardized incidence rate (ASIR) and crude mortality rate (CMR) of NPC has decreased, but the ASIR and crude incidence rate (CIR) increased in China (7). In 2020, there were 62,000 new cases of NPC in China, accounting for about 80% of the world’s NPC cases (8).

NPC poses a significant public health burden in endemic regions (9). In 2013, NPC incidence and mortality in China were also at high levels worldwide (10), which poses a great health burden in China (7). NPC remains a significant public health issue in China’s southern provinces, southern provinces including Guangdong, Guangxi, and Hainan had the highest years of life lost (YLL) rate in 2020 (11). Since 2005, the Chinese Ministry of Finance and the Ministry of Health have included rural cancer early diagnosis and treatment projects in the central subsidy for local special projects, and NPC has also been included in the scope of free screening (12). The NPC screening program can improve the long-term survival status of nasopharyngeal cancer patients (13). China has made great achievements in the prevention, control, diagnosis, and treatment of NPC in the past 30 years (14).

This study analyzes the medical expenditure and influencing factors of inpatients with NPC and aims to provide reference suggestions for reducing medical expenditure for NPC.

## 2 Materials and methods

### 2.1 Data source

The data used in this research was derived from one of the western area China hospitals. The data mainly includes the basic information and medical expenditure information of NPC inpatients, including age, gender, marital status, occupation, admission status, medical payment method, length of stay, surgical conditions, diagnosis and treatment of diseases, Surgical situation, blood transfusion conditions, treatment status, and readmission conditions, total medical expenditure, out-out-of-pocket costs, and the rate of out-out-of-pocket costs. According to Liao and Zhang (15), data for “total medical expenditure < 100 yuan,” “length of stay < 1 day,” and “length of stay > 90 days” were deleted. And we are removing the data on unreasonable medical expenditures. The sample includes inpatients from 2019 to 2023, with sample sizes of 2,489, 2,583, 3,058, 3,341, and 3,615.

### 2.2 Methods

#### 2.2.1 Data description method

The data on medical expenditure followed a positively skewed distribution, so it was described with medians and quartiles. Additionally, mean and standard deviation were also provided. Rank-sum test was performed to analyze the correlation between the characteristics of inpatients and the expenditure. A *p*-value less than 0.05 indicates significance.

#### 2.2.2 Random forest model

Random forest (RF) model is not very prone to overfitting (16), obtain predictions without obvious deviations (17), and provides a unique and essential feature for variable selection (18). Medical expenditure has a positively skewed distribution (19), therefore many researchers use the RF model to analyze the influence factors of medical expenditure.

Taloba et al. (20) use the d RF model to predict the medical expenditure. Wang and Shi (21) use the RF model to predict the medical expenditures of diagnosed diabetics and the assessment of its related factors. Kumagai and Jakovljević (22) use the RF model used to predict the medical out-of-pocket costs of hypertensive patients.

The sample is explained below into subsets: 80% of the data are used for training, and 20% of the data are used for testing. When an RF model randomly selects explanatory variables from among all the explanatory variables, the sample of the regression tree is split (22). At each internal node of the decision tree, entropy(E) is given by Equation (1).


(1) $$E=-\sum_{i=1}^{c}p_{i}\times \log(p_{i})$$

Where *c* is the number of unique classes, and *p_i_* is the prior probability of each class (23).

The RF model’s analysis aim is to rank the important factors of the dependent variables. The RF model used the two measurements to rank all variables: increase in mean squared error (%IncMSE) and increase in node purity(IncNodePurity), with larger values indicating greater importance of the variable. %IncMSE by randomly assigning values to each predictor variable, if the predictor variable is more important, the model’s prediction error will increase after its value is randomly replaced. IncNodePurity is measured by the sum of squared residuals, representing the impact of each variable on the heterogeneity of observations at each node of the classification tree, to compare the importance of variables.

## 3 Results

### 3.1 General description

The change in total medical expenditure, out-of-pocket cost, and the rate of out-of-pocket costs in 2019–2023 are shown in Table 1.

**Table 1:** Medical expenditure, out-of-pocket cost, and the rate of out-of-pocket cost of NPC inpatient 5 years.

Variables	*N*	M	P25	P75	Mean	SD	Min	Max
Total medical expenditure	15,086	15155.85	8278.1	32429.53	29014.58	35672.27	102.31	525277.8
2019	2,489	17402.92	9650.54	37970.2	36459.8	44341.92	104.79	222937.9
2020	2,583	16162.29	7636.88	32746.71	31175.3	38692.83	102.31	226635.6
2021	3,058	14833.13	8563.2	29320.59	26106.18	30613.24	120.26	194847.2
2022	3,341	15259.88	8948.97	36228.71	29050.6	35127.65	112.7	525277.8
2023	3,615	12855.77	7228.9	28641.85	24771.49	29807.51	326.57	285514.8
Out-of-pocket costs	15,086	7181.665	3311.28	15460.64	13139.68	17922.21	101.68	194715.4
2019	2,489	8102.29	3329.84	20495.14	17990.47	25729.96	104.79	183,842
2020	2,583	7359.64	2784.95	16604.67	15062.42	21720.81	102.31	158059.1
2021	3,058	7529.49	3879.03	15808.84	12637.4	15166.98	102.04	182322.8
2022	3,341	7859.68	4019.92	15651.97	12442.71	14096.3	112.7	194715.4
2023	3,615	5832.78	2675.26	11707.54	9494.994	11699.15	101.68	155073.4
The rate of out-of-pocket costs	15,086	0.474	0.318	0.658	0.503	0.242	0.006	1
2019	2,489	0.506	0.286	0.984	0.548	0.305	0.015	1
2020	2,583	0.509	0.319	0.686	0.534	0.264	0.011	1
2021	3,058	0.509	0.366	0.675	0.535	0.219	0.006	1
2022	3,341	0.471	0.336	0.642	0.488	0.204	0.020	1
2023	3,615	0.411	0.276	0.614	0.436	0.209	0.008	1

Total medical expenditure decreased from 9650.54 yuan in 2019 to 7636.88 yuan in 2020, then continuously increased from 7636.88 yuan in 2020 to 15259.88 yuan in 2022, but it declined to 12855.77 in 2023. From 2019 to 2023, total medical expenditure decreased by 26.13%. Out-of-pocket cost decreased from 8102.29 yuan in 2019 to 7359.64 yuan in 2020, then continuously increased from 7359.64 yuan in 2020 to 7859.68 yuan in 2022, but it declined to 5832.78 in 2023. From 2019 to 2023, the out-of-pocket was decreased by 28.01%. Additionally, the rate of out-of-pocket costs was continuously increased from 0.506 in 2019 to 0.509 in 2021, then which continuously decreased from 0.509 in 2021 to 0.411 in 2023.

### 3.2 Univariate analyses between the total medical expenditure and potential variables

It was found that the total medical expenditure was different among age, occupation, admission status, medical payment method, length of stay, surgical conditions, diagnosis and treatment of diseases, Surgical situation, blood transfusion conditions, treatment status, and readmission conditions. Detail information was listed in Table 2.

**Table 2:** Comparison of total medical expenditures among different information in NPC inpatient.

Variable	Classification	*N* (%)	Mean ± SD	M (Quartiles)	*p*
Age					<0.001
	≤40 years	4,344	28165.97 ± 34150.96	14969.83 (8068.07,31066.81)	
	40–50 years	4,387	28964.26 ± 35804.8	15048.81 (8292.3,31656.12)	
	50–60 years	4,435	28266.54 ± 36167.59	14591.81 (7835.3,30662.65)	
	60 years above	1920	32777.4 ± 37335.46	17069.31 (9616.325,44116.1)	
Gender					0.086
	Female	3,792	30277.51 ± 37345.47	15207.02 (8438.555,34907.88)	
	Male	11,294	28590.55 ± 35084.12	15137.3 (8225.85,31554.2)	
Marital status					0.141
	No spouse	1,026	29108.53 ± 33853.92	15805.83 (8563.2,33275.32)	
	Has a spouse	14,060	29007.72 ± 35802.49	15128.77 (8255.145,32313.8)	
Occupation					<0.001
	Unemployed retirees and other personnel	2,148	31612.1 ± 37437.19	16811.72 (9445.115,35864.45)	
	Farmer	5,124	30055.46 ± 37193.48	15563.08 (8580.56,31749.12)	
	Non-farmers	7,814	27617.99 ± 34061.45	14286.11 (7790.46,31713.63)	
Admission status					<0.001
	Other	985	33683.18 ± 38048.56	17744.46 (11041.74,40805.52)	
	Outpatient service	14,080	28643.25 ± 35403.72	14946.86 (8123.63,31695.89)	
	Emergency	21	58998.92 ± 64055.59	29893.2 (13771.95,99962.45)	
Medical payment method					<0.001
	Full self-pay	1,298	20353.81 ± 31753.21	10770.9 (1041.72,21540.88)	
	Urban and rural resident basic medical insurance	9,750	29663.07 ± 36069.93	15413.39 (8741.25,33033.42)	
	Urban employee basic medical insurance	3,923	30374.47 ± 35708.09	15824.86 (8570.28,37373.22)	
	Other insurance	115	25397.38 ± 28597.79	15629.6 (9131.48,31554.2)	
Length of stay					<0.001
	≤10 days	10,458	17410.2 ± 19522.78	11092.28 (6392.25,18106.92)	
	10–50 days	4,112	44205.3 ± 36823.51	27872.9 (18598.28,59057.59)	
	50–90 days	516	143150.9 ± 32878.39	139110.6 (124139.7,156182.1)	
Diagnosis and treatment of diseases					<0.001
	Number of disease diagnoses < 5	10,763	23757.51 ± 29640.28	13427.16 (7362.09,24314.9)	
	Number of disease diagnoses ≥5	4,323	42103.13 ± 44873.44	22113.25 (11530.57,58245.77)	
Surgical situation					<0.001
	Not undergoing surgery	14,192	28276.21 ± 35347.41	14473.4 (7930.335,30732.75)	
	Undergoing surgery	894	40735.95 ± 38657.13	26185.55 (17776.89,49865.05)	
Blood transfusion situation					<0.001
	Not blooding transfusion	14,863	28564.91 ± 34863.67	15048.7 (8222.41,31666.87)	
	Blooding transfusion	223	58,985 ± 64658.79	36075.7 (14935.77,84317.59)	
Treatment status					<0.001
	Death	10	49349.02 ± 51658.92	35304.68 (10243.27,60726.98)	
	Untreated	41	26061.1 ± 49546.61	14308.72 (8977.17, 21539.69)	
	Improved	10,353	29322.63 ± 35832.18	15622.34 (8572.26,32187.57)	
	Cured	256	72704.98 ± 49971.79	69047.96 (24565.96,119579.5)	
	Other	4,426	25748.37 ± 32256.31	13251.22 (7513.31,28529.85)	
Readmission status					<0.001
	Not readmitted	5,097	45068.45 ± 48356.46	20504.1 (9554.72,72531.93)	
	Readmission	9,989	20822.91 ± 23026.17	13772.46 (7577.88, 23351.93)	

### 3.3 The influence factors the medical expenditure of NPC inpatient with RF model

Total medical expenditure, out-of-pocket cost, and the rate of out-of-pocket costs were the output in the RF model, respectively. Total medical expenditures, out-of-pocket cost, and the rate of out-of-pocket payments (defined as *Y*, respectively), and the total medical expenditures, out-of-pocket cost were transformed into the new values (defined as *Y′*, respectively) with the transformation of *Y′*= ln(Y). Potential variables mentioned before were inputs in the RF model.

The data were randomly divided into the training dataset, which included 12,070 individuals, and the test dataset, which included 3,016 individuals, based on the proportion of eight-tenth and two-tenth, approximately.

Finding the optimal mtry using grid search and 10-fold cross-validation method. Then using training dataset to build the RF model in which the number of trees was defined based on the trend of the values of out-of-bag error rates with different numbers of trees. Finally, according to the results build an RF model.

Next, the ln-transformed medical expenditures of NPC in the dataset were analyzed based on the RF model built.

**Figure 1:**
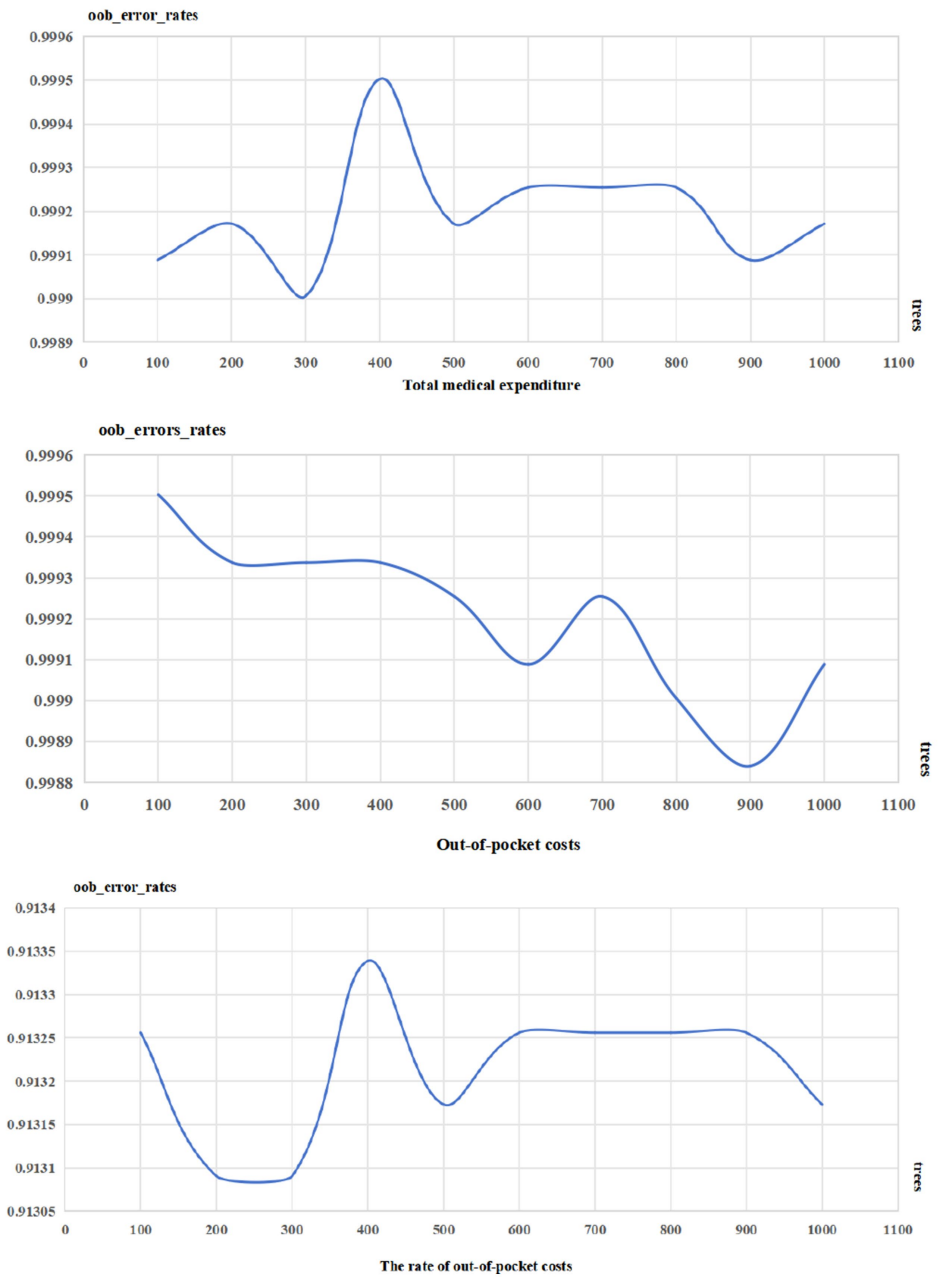
Trees and oob_error_rates.

According the results, when the total medical expenditures was *Y′*, the optimal parameters for the model were 3 of mtry and 300 trees; when the out-of-pocket costs was *Y′*, the optimal parameters for the model were 3 of mtry and 900 trees; when the rate of out-of-costs was *Y*, the optimal parameters for the model were 3 of mtry and 200 trees. Detail information was listed in Table 3 and Figure 1.

**Table 3:** Model situation of different mtries.

Y	mtry	RMSE	Rsquared	MAE
Total medical expenditure	1	1.065	0.405	0.783
	2	0.951	0.443	0.718
	3	0.929	0.453	0.701
Out-of-pocket costs	1	1.113	0.331	0.856
	2	1.023	0.366	0.788
	3	1.002	0.378	0.770
The rate of out-of-pocket payments	1	0.205	0.429	0.175
	2	0.183	0.446	0.153
	3	0.180	0.451	0.144

**Figure 2:**
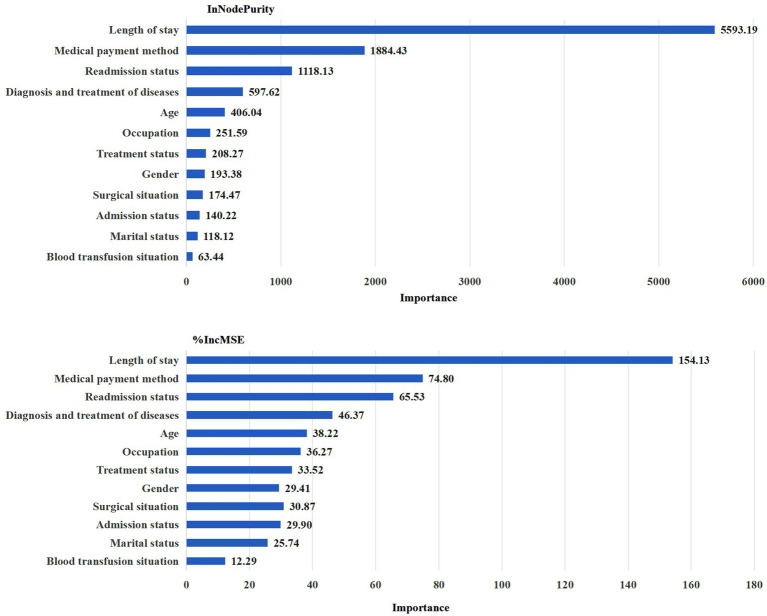
Total medical expenditure.

According to IncNodePurity and %IncMSE, the top five influence factors in total medical expenditure shown in Figure 2 that the top five influence factors were: Length of stay > Medical payment method > Readmission status > Diagnosis and treatment of diseases > Age.

**Figure 3:**
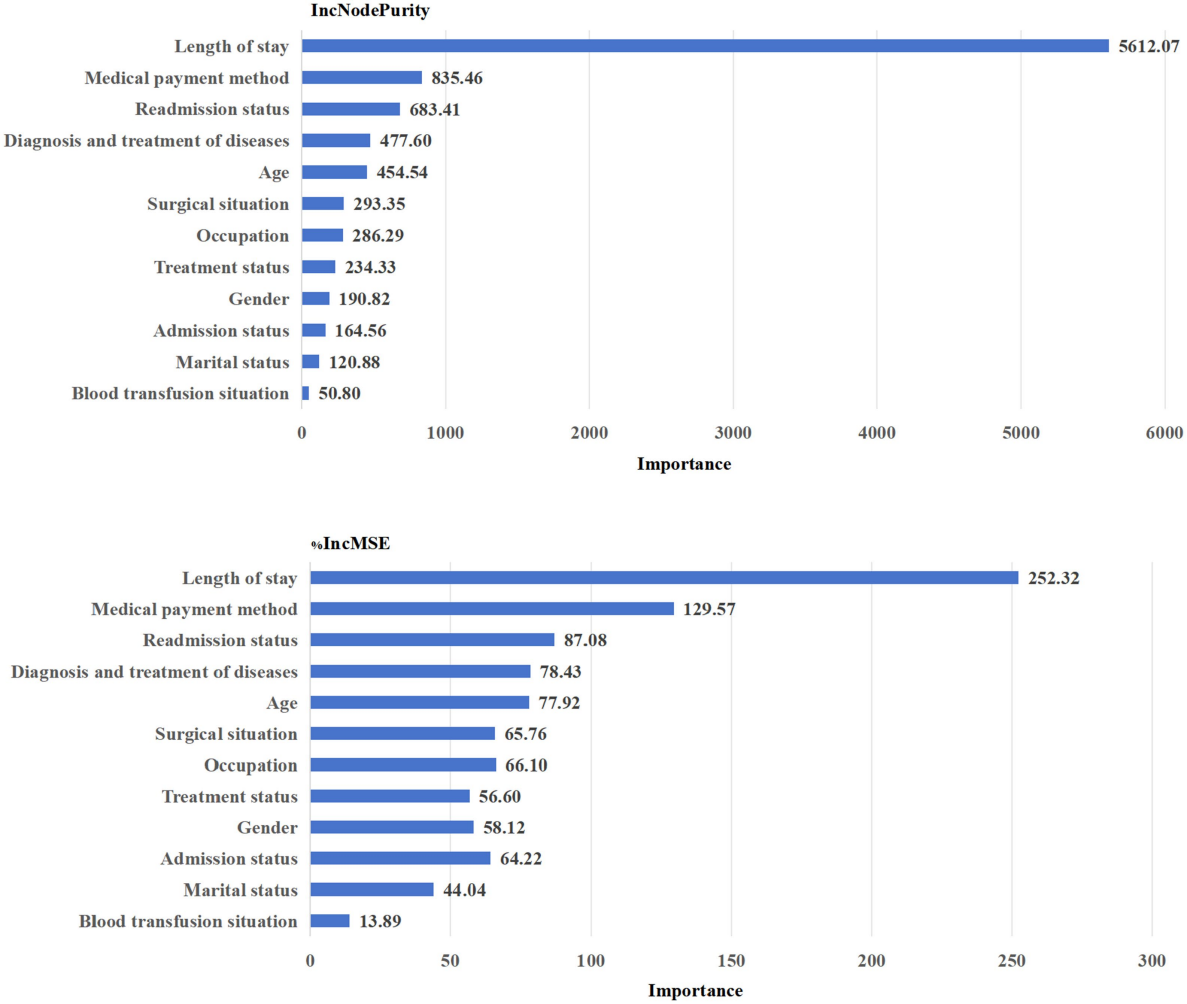
Out-of-pocket costs.

According to IncNodePurity and %IncMSE, the top five influence factors in the out-of-pocket costs shown in Figure 3 that the top five influence factors were: Length of stay > Medical payment method > Readmission status > Diagnosis and treatment of diseases > Age.

**Figure 4:**
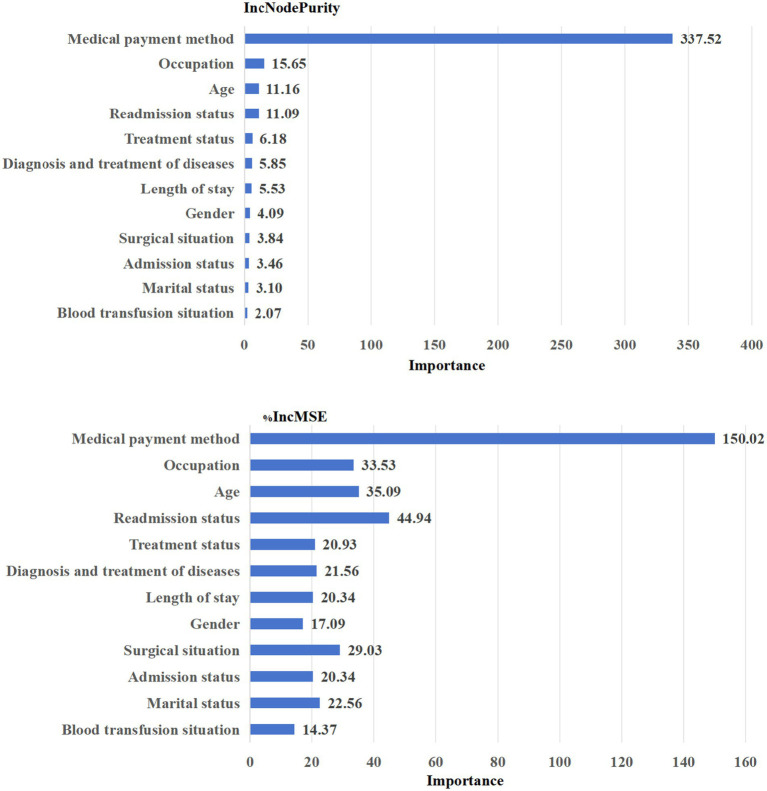
The rate of out-of-pocket costs.

According to IncNodePurity and %IncMSE, the top five influence factors in the out-of-pocket costs shown in Figure 4 that the top five influence factors were: Medical payment method > Occupation > Age > Readmission status > Treatment status.

**Figure 5:**
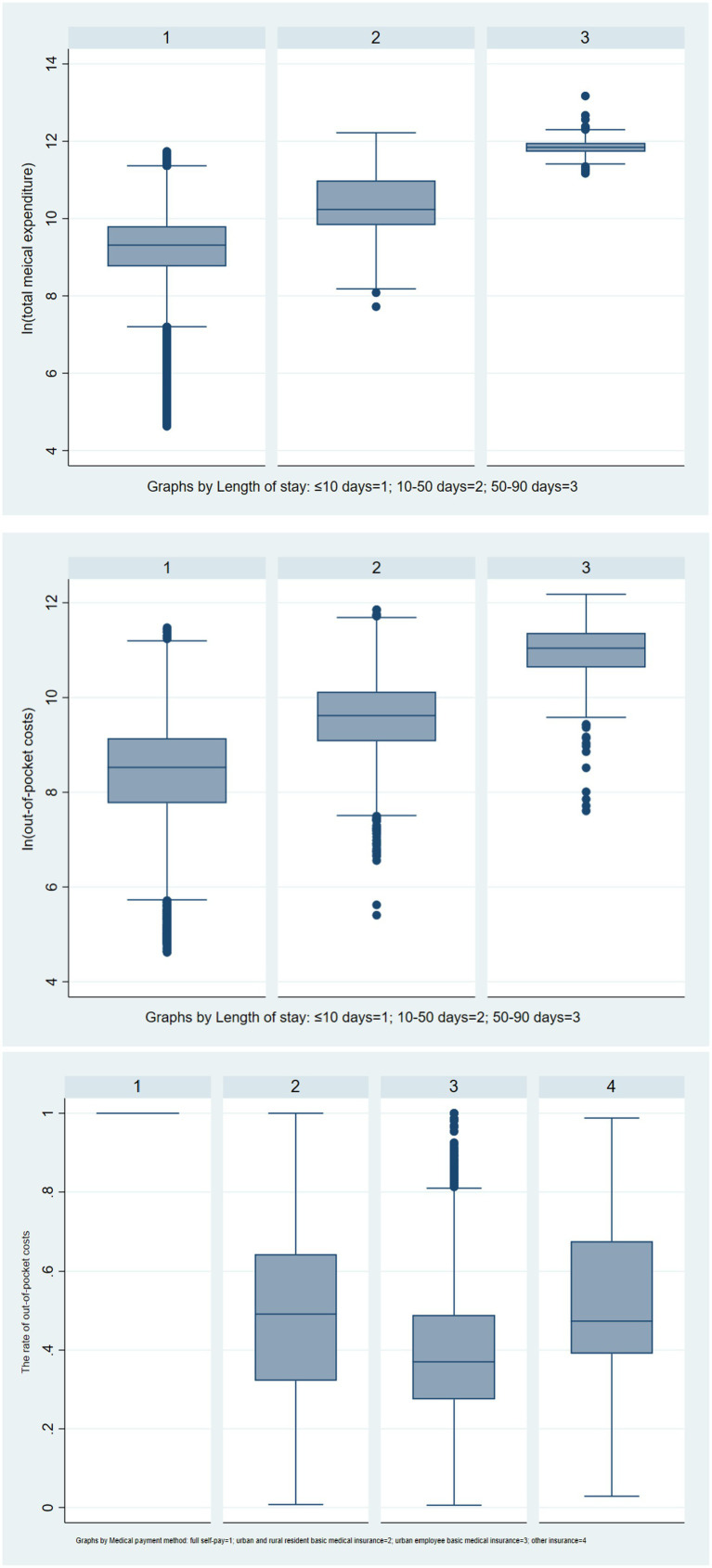
Boxplots of total medical expenditure and out-of-pocket costs varying with length of stay and the out-of-pocket costs varying with medical payment method.

Detailed information on total medical expenditure, out-of-pocket costs the length of stay, and the rate of out-of-pocket costs are listed in Figure 5.

About In(total medical expenditure), when the length of stay ≤ 10 days or between 50 and 90 days, the number of outliers is more than the length of stay between 10 and 50 days’ outliers. The median total medical expenditure varies among different groups, with the highest being between 50–90 days of the length of stay, the In(total medical expenditure) close to 12, followed by between 10–50 days of the length of stay, In(total medical expenditure) slightly higher at 10, and the lowest being ≤ 10 days of the length of stay, In(total medical expenditure) close to 10.

The median out-of-pocket costs vary among different groups, with the highest being between 50–90 days of the length of stay, the In(out-of-pocket costs) around 11, followed by between 10–50 days of the length of stay, In(out-of-pocket costs) slightly lower at 10, and the lowest being ≤ 10 days of the length of stay, In(out-of-pocket costs) slightly higher 9.

About the rates of out-of-pocket costs, when the medical payment method was urban employee basic medical insurance, there were some outliers. The median rates of out-of-pocket costs vary among different groups, with the highest being full self-pay, the rates of out-of-pocket costs were 1, followed by urban and rural resident basic medical insurance, the rates of out-of-pocket costs were close to 0.5, then other insurance, the rates of out-of-pocket costs was slighter higher 0.4, and the lowest was urban employee basic medical insurance, the rates of out-of-pocket costs was lower 0.4.

**Figure 6:**
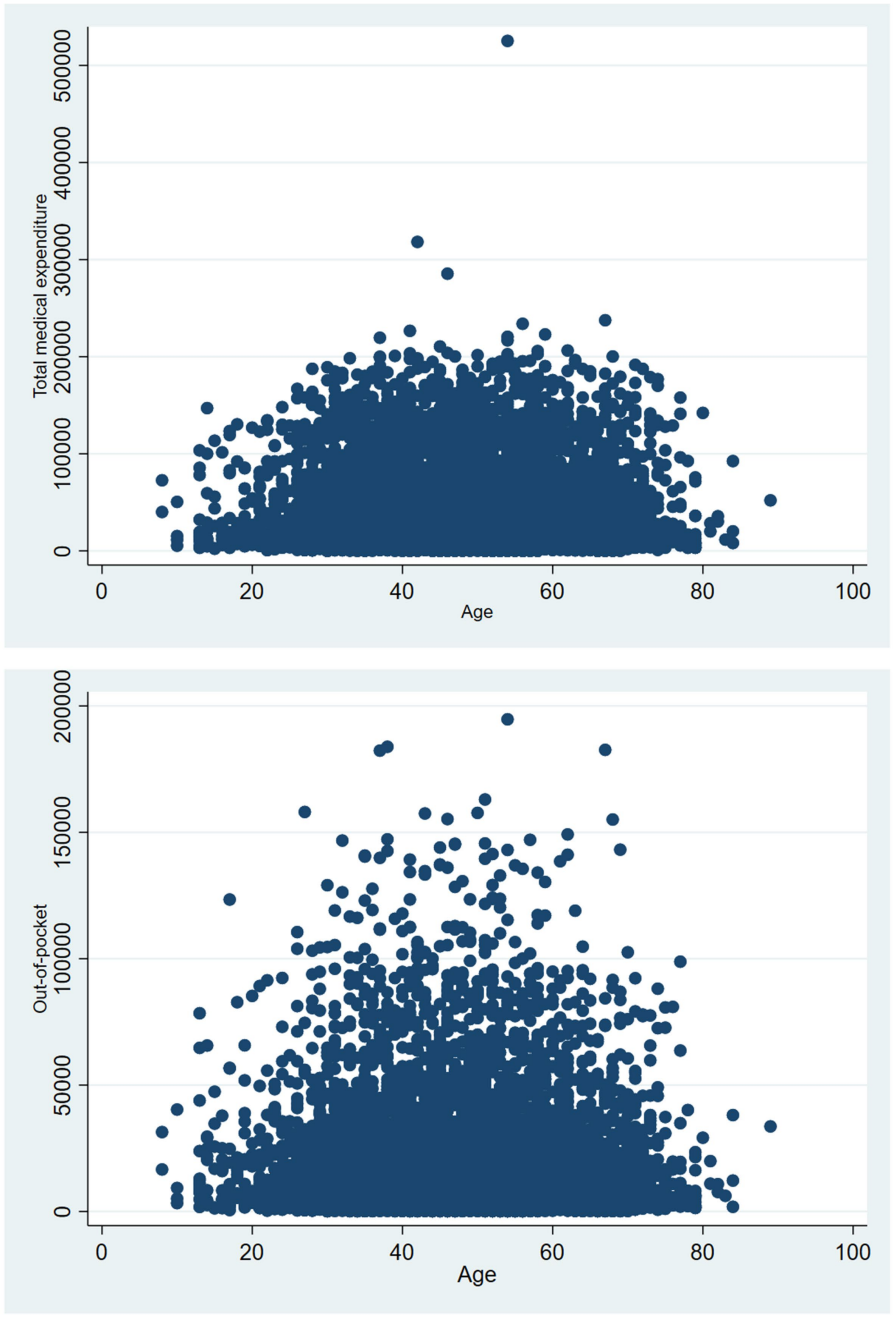
Density scatter plots of total medical expenditure varying with age and out-of-pocket costs varying with age.

It is evident that the majority of NPC inpatients are aged between 40 and 60 years, and inpatients exhibiting a total medical expenditure ranging from 0 to 100,000 yuan demonstrate the highest density, and those with medical out-of-pocket costs between 0 and 50,000 yuan also exhibit the highest density. Detailed information is listed in Figure 6.

## 4 Discussion

Total medical expenditure, out-of-pocket costs, and the rates of out-of-pocket costs from 2019 to 2023 among NPC inpatients in one of the western area China hospitals decreased and were consistent with each other. We further found the length of stay was an important factor in total medical expenditure and out-of-pocket costs, and the medical payment method was an important factor in the rates of out-of-pocket costs. Then, we found that with longer stays, the total medical expenditure and out-of-pocket costs were higher. Different insurance affects the rate of out-of-pocket costs differently, compared with other insurance, the urban employee basic medical insurance reduces the NPC inpatient’s medical expenditure burden the best. The majority of NPC inpatients are aged between 40 and 60 years, and inpatients exhibiting a total medical expenditure ranging from 0 to 100,000 yuan demonstrate the highest density, and those with medical out-of-pocket costs between 0 and 50,000 yuan also exhibit the highest density.

About NPC inpatients’ total medical expenditure, out-of-pocket costs, and the rates of out-of-pocket costs were decreased. We speculate that there are the following reasons: (1) the government increases health expenditure on NPC. Death and cancer mortality rates among rural residents were higher and increased faster than among urban residents (24). NPC patients with high socioeconomic status had better overall survival compared with those who had low and medium socioeconomic status (25). Women having financial concerns after diagnosis were more likely to reduce their non-medical expenses and even quit treatments (26). It can be seen that the economic level has a significant impact on health. In order to provide all citizens with equal access to basic healthcare with reasonable quality and financial risk protection, China launched a major healthcare reform in 2009 (27). From 2015 to 2022, the average annual growth rate of total health expenditure is 8.32% in China (28). The increase in government medical expenditure will help reduce personal medical expenditure. (2) Medical insurance develop. With the development of medical, the proportion of personal payments to total health expenditure has significantly decreased (29). The economic burden of diseases among the population with basic medical insurance for urban employees, public medical insurance, and commercial medical insurance has decreased by 5% or 4% (30). The cost of treating malignant tumors is a heavy personal burden (31). In 2017, the treatment of NPC with rituximab was on the medical insurance drug list, which significantly improved the level of medical insurance drug protection and greatly reduced the economic burden (32). (3) Medical technology improvement. In this case-matched study, we observed that endoscopic nasopharynx ectomy resulted in significant decreases in medical costs and length of hospital stay for recurrent NPC, as compared with intensity-modulated radiotherapy (33).

Our research findings are somewhat consistent with those of others. The length of stay was an important factor in total medical expenditure and out-of-pocket costs. Other research found that the length of stay is one of the main factors affecting the total hospitalization cost for malignant tumors (34).

We found that the medical payment method has the greatest impact on the rates of out-of-pocket costs. Urban employee basic medical insurance provides the best level of protection for NPC patients. Next are other medical insurance and rural resident basic medical insurance. The full self-pay medical payment method imposes a heavy burden on medical expenditure. Because the reimbursement ratio for urban employee basic medical insurance, the level of protection is also higher. Other medical insurance and rural resident basic medical insurance for residents can ensure the basic health of the people. Lack of insurance will increase the vulnerability to disease risks. In 2023, the current medical insurance coverage rate is over 95% in China. That is to say, there are still some people who do not have insurance, and attention should be paid to this group of people.

We found that the total medical expenditure of NPC inpatients shows no gender disparities. But NPC shows gender disparities, with higher rates in males (4, 35). In Asia, males below 50 years old are more prone to NPC and are often diagnosed at the late stage (36). That is, reducing the incidence rate of male NPC will help reduce the medical expenditure burden of NPC in China. Males’ biological factors and high-risk behaviors caused a higher NPC incidence rate. Cigarette smoking was associated with increased risk of NPC, especially for young smokers (37). Among the high-risk population for NPC, the EB virus infection rate in males is 9.50%, which is higher than that in females at 5.83%; in the population with a family history of inheritance, the EB virus infection rate in males is 24.75%, which is higher than the 18.08% rate in females (38). Therefore, to control the occurrence of NPC and reduce the medical expenditure of NPC, reduce smoking, and attention to oral hygiene to reduce the spread of EB virus.

We found the largest number of NPC in patients aged between 50 and 60 years. The result is the same as Chang et al.’ result, NPC incidence exhibits a single peak at approximate ages 45–59 years in high-risk populations (39). The effect of age on survival is marked in NPC, five-year survival rates were 72% in the youngest age group (15–45 years) and 36% in the oldest group of patients (65–74 years) (40). We further found that there is an age difference in the total medical expenditure for NPC inpatients. According to our research findings, the total medical expenditure and out-of-pocket costs of NPC inpatients are mainly between the ages of 40–60. NCP has a prognosis that is gloomy after metastases are diagnosed, with a fatality rate of 91% within a year of the first metastasis (41). NPC is a rare cancer in developed areas, which may be related to early prevention, screening, diagnosis, and later treatment in developed regions (42). NPC relatively common malignant tumor, screening in high-risk areas can significantly improve early diagnosis rates and reduce treatment costs (43). China has carried out several screening programs in high-incidence areas (4). In the future prevention and treatment of NPC, it is recommended to strengthen the screening of high-risk populations to detect early asymptomatic NPC patients and enhance the tertiary prevention of NPC.

## 5 Conclusion

Total medical expenditure, out-of-pocket costs, and the rates of out-of-pocket costs were decreased. Length of stay was an important factor in total medical expenditure and out-of-pocket costs, and the medical payment method was an important factor in the rates of out-of-pocket costs. There are suggestions to reduce the burden of NPC patients’ medical expenditure. First, we can reduce the length of stay by improving the level of medical technology. Second, increase government medical expenditures and improve the level of medical security. Third, promote smoking bans and strengthen screening in high-risk areas. This study has several limitations: (1) Existing data has limitations. The main treatment for NPC patients is Simultaneous chemoradiotherapy. The long radiotherapy cycle and the patients’ unwillingness to be hospitalized for a long period lead to the fact that some patients are not hospitalized at one time, and there are records of multiple hospitalizations. Radiotherapy costs are not charged according to the number of days. For patients who have been hospitalized numerous times, different parts of radiotherapy costs are charged in different hospitalization records. The above indicates that some data show patients have a higher out-of-pocket cost rate despite having medical insurance. In special circumstances where only out-of-pocket costs are collected, the out-of-pocket costs rate for medical insurance participants may be as high as 100%. This data situation may potentially lead to some bias in findings. (2) Due to the availability of data, we were unable to incorporate additional influential factors into the analysis. Then we could not explore additional factors and their mechanisms that affect the medical expenses of NPC. (3) The current data were collected from NPC patients across different periods rather than through longitudinal tracking of the same individuals, resulting in the loss of some influencing factors and limiting the exploration of their influencing mechanisms.

## Data Availability

The datasets presented in this article are not readily available because the dataset is private. Requests to access the datasets should be directed to zhupinghua@gxmu.edu.cn.
